# Urinary nephrin linked nephropathy in type-2 diabetes mellitus

**DOI:** 10.6026/973206300181131

**Published:** 2022-12-31

**Authors:** Hareesh Rangaswamaiah, Pallavi Somashekar, Raghav Gutlur Nagarajaiah Setty, Veluri Ganesh

**Affiliations:** 1 Department of General Medicine, Akash Institute of Medical Sciences and Research Centre, Bangalore - 562110, Karnataka, India; 2 Department of General Medicine, Akash Institute of Medical Sciences and Research Centre, Bangalore - 562110, Karnataka, India; 3 Department of General Medicine, Rajarajeswari Medical College and Hospital, Bangalore - 560074, Karnataka, India; 4 Department of Biochemistry, Akash Institute of Medical Sciences and Research Centre, Bangalore - 562110, Karnataka, India

**Keywords:** Urinary ACR, type 2 diabetes mellitus, nephropathy and nephrin

## Abstract

Diabetic nephropathy is a one of the risk factor for end stage renal disease in patients with type 2 diabetes mellitus. Urinary micr oalbumin currently marker using for diagnosis of nephropathy in type 2 diabetes mellitus due to its larger variability and less specificity. Urinary nephrin is podocyte protein can be act as a early predictable and prognostic marker than micro albumin for nephropathy in type 2 diabetes mellitus. This case-control prospective observational study included 60 type 2 diabetes mellitus and 30 controls after applying criteria. Basic biochemical, clinical and experimental data were measured and recorded. There was a significantly elevated level of urinary nephrin in type 2 diabetes mellitus patients when compared to controls. The urinary nephrin significantly elevated in normo albuminuria group only when compared to urinary ACR and it is positive association with kidney damage. This study concluded significantly elevated levels of urinary nephrin might be used for early diagnosis and prognostic marker for nephropathy in type 2 diabetes mellitus.

## Background:

Type 2 diabetes mellitus (T2DM) is a complex metabolic disorder characterized by hyperglycemia due to insulin resistance in target tissues including the liver and skeletal muscles. This leads to multisystem failure [1]. The prevalence in 2015, it was projected that 415 million individuals living with diabetes globally and estimated to rise up to 650 million by 2040. There were 65 million Indian people living with diabetes by 2013 and estimated to reach 109 million people by 2030 [2,3]. This persistent metabolic condition damages the metabolism of electrolytes, water, protein, and carbohydrates. Over time, hyper glycemia destroys the blood vessel's basement cell membrane, leading to the malfunction and failure of a number of organ systems, particularly the heart, blood vessels, kidneys, eyes, nerves, and kidneys [4]. Insulin resistance and decreased insulin production caused by pancreatic beta-cell dysfunction are the two primary disorders of type 2 diabetes mellitus [5]. However, it is evident that the disease process is heterogeneous and consists of other pathogenic components. The interaction of genes and environment has a significant role in determining insulin resistance and beta-cell malfunction. The first gene discovered was peroxisome proliferate-activated receptor gamma (PPARG) [6]. 33 of the 53 loci that have been connected to insulin and glucose levels are linked to type-2 diabetes. Most loci are connected to -cell function, however some are linked to obesity and insulin resistance. Additional environmental elements include calorie intake increases and decreased energy expenditure Increased intakes of saturated fat in the diet, in particular, are crucial for the emergence of obesity, insulin resistance, beta-cell dysfunction, and glucose intolerance. Furthermore, the decline in glucose tolerance with age is partly caused by an age-related decrease in the reactivity of cells to carbohydrates [7 8]. Glomerular hyper filtration, thickening of the glomerular basement membrane, and an expansion of extracellular matrix in masangial zones are only a few examples of the structural and functional alterations in the glomerulus that lead to DN [9]. Recent research has shown that proximal tubular cell atrophy and tubule-interstitial fibrosis is just as critical as glomerulosclerosis in terms of the prognosis of the kidneys [10-11]. DN is a clinical syndrome characterized by persistent albuminuria (or albuminuria excretion rate of >300 mg/day or 200 g/min), gradual GFR decline, and hypertension that has been observed at least twice during a 3- to 6-month time frame. The morbidity and mortality associated with DN will undoubtedly be decreased by improved understanding of the risk factors for diabetic nephropathy, early identification of patients at risk for diabetic renal disease, and early beginning of suitable treatment [12 13]. According to recent research, DN risk factors include hyperglycemia, progressive microalbumin levels in urine, elevated serum lipid levels, blood pressure, glycosylated haemoglobin, smoking, inactivity, advanced age, high levels of insulin resistance, and origin (amount and source) of dietary protein [14,15]. The connections between metabolic and hemodynamic pathways, which are frequently disrupted in the context of diabetes, are what cause the origin and evolution of diabetic nephropathy. Diabetes is associated with metabolic and hemodynamic disorders that interact with pathways that lead to ROS production. The results of these result in structural and functional modifications that clinically emerge as diabetic nephropathy, which is defined by rising albuminuria and deteriorating renal function [16]. Over time, it has become clearer and clearer that hyperglycemia is not the only factor contributing to DN. The development of DN involves a number of pathophysiological pathways, including the hemodynamic pathway, metabolic pathways, inflammation pathway, and other alternation pathway. Due to its limited sensitivity and high variability, micro albuminuria, the current gold standard for early identification of diabetic nephropathy, has its limitations. It also has a number of disadvantages. The plasma concentrations of atrial natriuretic peptide, arginine vasopressin, angiotensin II, aldosterone, fasting blood glucose, glycated haemoglobin, and mean arterial blood pressure can all have an impact on how much of it is excreted. There is a 47% inter-individual variance. These elements make it impossible to employ albuminuria as the traditional biomarker for the early identification of DN [17]. Therefore, there is a need for biomarkers that can detect the presence of diabetic nephropathy with high levels of sensitivity, specificity, and prediction. Additionally, early detection of diabetic nephropathy prior to the onset of micro albuminuria might improve prognosis and allow for earlier treatment. The trans-membrane protein nephrin, which belongs to the immunoglobulin superfamily, is a crucial part of the slit diaphragm that lies between the foot processes of the podocytes. Its modifications result in the slit diaphragm's size-selectivity being limited [18]. The glomerular basement membrane, the podocyte layer, and the fenestrated endothelium are the three main parts of the glomerular capillary wall. Specialized epithelial cells called podocytes create a network of inter digitating foot processes [19]. According to a recent study by Jim et al.,all type 2 diabetic patients with micro albuminuria and macro albuminuria had nephrinuria. Nephrinuria in normo albuminuric individuals, which comes before micro albuminuria, may be caused by dysregulation of 26 nephrin in podocytes in DN. According to a survey, 54% of type 2 diabetic patients with normo albuminuria had nephrinuria [20]. It has been demonstrated that nephrinuria develops in type 1 DM patients before micro albuminuria. Additionally, several normo albuminuric type 2 DM patients reported having nephrinuria. Nephrinuria is a biomarker for early glomerular injury and is associated with podocyte damage [21]. Nephrinuria, a biomarker of DN in other phases of DM as well, is favorably connected with albuminuria in albuminuric individuals and negatively correlated with GFR. Diabetes patients with normo albuminuria may have higher levels of nephrinuria, demonstrating the usefulness of nephrinuria in diagnosing DN before micro albuminuria develops [22]. Since urine nephrin levels appear to rise before micro albuminuria, even if additional research is needed, they are still likely to be a reliable biomarker of early diabetic kidney impairment.

## Materials and Methods:

To investigate urine indicators in the early diagnosis of diabetic nephropathy in patients with type 2 diabetes mellitus, a case-control prospective observational study was conducted. The study included a total of 90 participants, 30 healthy controls, and 60 patients who were diagnosed with T2DM according to American Diabetes Association (ADA) criteria between March 2017 and February 2018 and who visited the endocrinology, metabolism, and nephrology outpatient departments at Akash Institute of Medical Sciences and Research Centre, Bangalore Rural, Karnataka. Thirty wholesome controls made constituted Group 1. Based on the albuminuria levels, the patients were split into two groups of thirty each: type 2 diabetes mellitus patients with normo albuminuria (Group 2) and type 2 diabetic mellitus patients with micro albuminuria (Group3). After presenting the study to the subjects in their native tongue, their informed consent was obtained. After receiving approval from the institutional thesis protocol approval committee and institutional ethics committee, the study was carried out.

## Collection of urine sample:

Random spot urine samples were taken to check for albuminuria in all the groups of study subjects. The patient was placed in Group 2, or type 2 DM with normoalbuminuria, if the UACR levels were less than 30 mg/g creatinine and type 2 DM with microalbuminuria, if the UACR levels above 30 but fell short of 299 mg/g creatinine were considered.

## Statistical analysis:

Kolmogorov-Smirnov test was used to assess the distribution of the data. For categorical data, continuous variables are expressed as mean and standard deviation. ANOVA was used to assess differences between the three groups under investigation, and it was followed by post hoc multiple testing using Tamhane's or Bonferroni's tests, if necessary. The correlations between the markers were examined using Pearson's correlation analysis. Microsoft Excel spreadsheets, SPSS software for Windows version 16 (SPSS Inc., Chicago, IL, USA) was used. Statistics were deemed significant at P 0.05. Table 1(see PDF) displays the demographic and medical traits of the T2DM cases and controls. The age of the study participants was, respectively, 43.90 ± 8.05 and 46.05 ± 4.75 years for controls and T2DM cases (p 0.001*). The mean FBS concentration was observed significantly elevated in T2DM patients (150.89 ± 53.08) when compared to controls (89.98 ± 9.84) respectively; P value 0.001**. The serum creatinine (0.79 ± 0.15 and 3.90 ± 3.12) and serum urea (20.65 ± 6.01 and 49.45 ± 28.71) were significantly increased in patients with T2DM when compared to controls respectively; P value 0.001**. Urinary ACR also significantly elevated in T2DM patients (45.19 ± 24.89) when compared to controls (4.96 ± 3.01) respectively; P value 0.001**. The T2DM patients (2.01 ± 1.51) showed a significantly elevated levels of urinary nephrin when compared to controls (0.68 ± 0.12) respectively; P value 0.001**. Table 2(see PDF) displays the demographic and medical traits of the study groups. The age of the study participants was, respectively, 43.90 ± 8.05, 45.38 ± 5.57 and 46.05 ± 4.75 years for group 1,2 and 3 (p 0.05*). The mean FBS concentration was observed significantly elevated in group 2 and 3 (140.30 ± 50.11 and 161.48 ± 54.47) when compared to group 1 (89.98 ± 9.84) respectively; P value 0.001**. The serum creatinine (0.79 ± 0.15, 0.94 ± 0.39 and 6.64 ± 1.66) and serum urea (20.65 ± 4.99, 22.53 ± 6.98 and 76.38 ± 11.55) were significantly increased group 3 and 2 when compared to group 1 respectively; P value 0.001**. Urinary ACR also significantly elevated in group 2 and 3 (10.56 ± 7.92 and 83.76 ± 39.03) when compared to group 1 (4.96 ± 3.01) respectively; P value 0.001**. The group 2 and 3 subjects (0.68 ± 0.12) showed a significantly elevated when compared to group 1 (2.01 ± 1.51) respectively; P value 0.001**. Table 3(see PDF) shows the pearson correlation urinary ACR with other parameters of the study, there was a significantly positive correlation between the urinary ACR and FBS, urea, creatinine and urinary nephrin (r = 0.449, 0.464, 0.246 and 0.855) respectively P value is 0.001**.Table 4(see PDF) shows the pearson correlation urinary nephrin with other parameters of the study, there was a significantly positive correlation between the urinary nephrin and FBS, urea, creatinine and urinary ACR(r = 0.522, 0.271, 0.110, 0.855) respectively P value is 0.001**. Figure 1(see PDF) represents the urinary ACR levels in T2DM cases and controls. The T2DM with microalbuminuria patients showed significantly elevated levels of urinary ACR when compared to T2DM with normoalbuminuria and controls. Figure 2(see PDF) represents the urinary nephrin levels in T2DM cases and controls. The T2DM with micro and normoalbuminuria patients showed significantly elevated levels of urinary nephrin when compared to controls. Figure 3(see PDF) represents scatter plots of urinary nephrin and urinary ACR. There was a significantly positive association between urinary nephrin and urinary ACR levels.

## Discussion:

Diabetic nephropathy is one of the most common conditions worldwide in T2DM. However, it has become increasingly evident over the years that hyperglycemia by itself is not the sole cause of DN. Several pathophysiological pathways including hemodynamic pathway, metabolic pathways, inflammation pathway and other alternation pathway are involved in the development of DN. Persistent micro albuminuria (30-299 mg/day), which is validated on at least two times three to six months apart, is currently the gold standard test for the early diagnosis of diabetic nephropathy. However, it has drawbacks, including low sensitivity, variable plasma concentration caused by a number of variables, and a significant inter-individual variation of 47% [23]. To lessen the burden of chronic renal disease in T2DM, innovative biomarkers for the early detection of DN and progression to ESRD must be found. The current study therefore sought to determine the link between urinary Nephrin in T2DM patients. In the current investigation, it was discovered that FBS, urea, creatinine levels were rising in the controls, T2DM patients with normo albuminuria, and T2DM patients with micro albuminuria respectively; P 0.001**. It is recommended that the urine biomarkers be reported with creatinine adjusted in the situation of renal disease without AKI. The first DN sign now used in clinical practice is micro albuminuria. However, a significant amount of renal damage happens before micro albuminuria develops or in a non-albuminuric condition [24]. Therefore, finding new biomarkers is necessary for DN early diagnosis. In light of this, the current investigation calculated the amounts of urinary nephrin levelsin the urine of healthy controls and T2DM patients with normo- and micro albuminuria. Correcting for age, partial correlation was conducted. A significant positive connection was found between albuminuria, the current gold standard, and the examined biomarkers urinary nephrin [25]. These results show that these biomarkers represent renal damage, either glomerular or tubular or both, and that they increase proportionally to the degree of renal damage. As a result, they can be used to track the development of DN as well [26]. The T2DM patients with normoalbuminuria shown significantly elevated levels of urinary nephrin and progressively elevated in T2DM patients with microalbuminuria might be useful for early detection and progression of nephropathy in patients with type 2 diabetes mellitus.

## Conclusion:

This study concludes significantly elevated levels of urinary nephrin levels in type 2 diabetes mellitus patients might be used for early diagnosis and progression of nephropathy.
